# Medical Dots from Louisville—Treatment of Pneumonia

**Published:** 1900-07

**Authors:** Robt. W. Skipper

**Affiliations:** Louisville Ky.; Lovelady, Texas


### Medical Dots From Louisville—Treatment of Pneumonia.

LOUISVILLE, KY., May 30, 1900.

*Editors Texas Medical Journal, Austin, Texas:*

It might interest the medical profession of Texas to read a few lines from the great medical center where thousands of them have taken their degrees, and especially from that renowned teacher of medicine, Joseph B. Marvin, who has helped hundreds of them through the "green room." He is still here in the Kentucky University, lecturing to a large and intelligent class. I believe his treatment of pneumonia will interest the profession, so I will give the main features, as just imparted to the students.

He would have a room with as much air as possible; not hot air, but cool. Our rooms, he says are kept too hot and too dry; would have a temperature of 60° to 65°, and put patient in open window, when dispnœa is great. The air must be changed often. He would not feed in the early stage of pneumonia, for feeding entails too much work upon the lungs.

"Make your diagnosis and let the patient alone. Do not examine him again." He would have no one talk with patient, "no, not even under the guise of religion."

For shock, early in disease, give morphia hypodermatically, then discontinue its use. To emphasize this point, he says: "I wish every doctor could have his hypodermic syringe with him to give this first dose, and that somebody would throw it away after that, so that he could not use it again." The morphia will slow respiration and relieve pain with shock. The ice bag is a favorite with him, applied direct to affected side. He insists upon its use whenever the prejudices of patient and friends can be overcome. "The poultice is a nuisance; let it be made, but put it upon the bed-post." The ice, he says, relieves pain and improves respiration, and your patient will "holler" for it. When pain holds on he advocates counter-irritation. No cough mixtures are to be used, and if any are given they must be the most simple. Syrup hydriodic acid with Brown mixture and muriate of ammonia may be used. "Do not use opiates in any cough mixture." Calomel he would use early, giving from five to twenty grains. He says, "I would hate to treat a case early without calomel."

Creosote with peptonoids, three to five m. of the former, with a tablespoonful of the latter; salicylates of soda and ammonia are good.

All these drugs and agents in the first stage, not to abort the disease, but to relieve shock and destroy the toxic agent that may be in the lungs. He believes that the inhalation of chloroform, either with or without creosote, menthol or terabine would aid greatly in meeting the same indications. Has not used it in this way, but others have. Must not anæsthetise. Quinine he would use for two or three days.

Dr. Marvin says: "The best stimulant is strychnia, used hypodermatically. Throw other heart tonics out of doors and give strychnia. You may bleed early by opening a vein. To remove blood from right side to left, use nitrates, nitroglycerine, 1-50 gr., and repeat in twenty minutes if necessary. It lowers tension and dilates the vessels. To be given only for a short while—a few hours or perhaps a few days. Give it and strychnia separately. Digitalis is good for valvular troubles, but nothing else. It slows the pulse, contracts the left ventricle, increases tension and puts more work upon the right heart. It is one of the worst agents you can give here."

Dr. Marvin uses amorphous digitalin, as made by Merck, not the digitalin of the pharmacopœia, and gives one-half grain doses. Has given as much as two grains. He believes this has a decided advantage over other preparations.

Whisky, he says, "is a pontoon bridge to tide the patient over an emergency." Would not give brandy, but whisky can be given for its effect. If pulse is slower give more in twenty to thirty minutes. If more rapid, do not give, or if restless. Give for rapid heart, and when there is lividity, and if doing good give more. Some, he says, may require but a teaspoonful, while others will require large quantities. Do not give with sugar, egg or milk, but with a little water or crushed ice.

Later in case, feed with pure milk, right from the cow. Give a little soda bicarb. and then the milk. During convalescence give the iodides, and exercise the respiratory organs by talking.

Lovelady, Texas. ROBT. W. SKIPPER.

